# Cognitive spare capacity: evaluation data and its association with comprehension of dynamic conversations

**DOI:** 10.3389/fpsyg.2015.00597

**Published:** 2015-05-06

**Authors:** Gitte Keidser, Virginia Best, Katrina Freeston, Alexandra Boyce

**Affiliations:** ^1^National Acoustic LaboratoriesSydney, NSW, Australia; ^2^Department of Speech, Language, and Hearing Sciences, Boston UniversityBoston, MA, USA; ^3^Department of Audiology, Macquarie UniversitySydney, NSW, Australia

**Keywords:** cognitive spare capacity, working memory capacity, updating, speech comprehension, dynamic speech test

## Abstract

It is well-established that communication involves the working memory system, which becomes increasingly engaged in understanding speech as the input signal degrades. The more resources allocated to recovering a degraded input signal, the fewer resources, referred to as cognitive spare capacity (CSC), remain for higher-level processing of speech. Using simulated natural listening environments, the aims of this paper were to (1) evaluate an English version of a recently introduced auditory test to measure CSC that targets the updating process of the executive function, (2) investigate if the test predicts speech comprehension better than the reading span test (RST) commonly used to measure working memory capacity, and (3) determine if the test is sensitive to increasing the number of attended locations during listening. In Experiment I, the CSC test was presented using a male and a female talker, in quiet and in spatially separated babble- and cafeteria-noises, in an audio-only and in an audio-visual mode. Data collected on 21 listeners with normal and impaired hearing confirmed that the English version of the CSC test is sensitive to population group, noise condition, and clarity of speech, but not presentation modality. In Experiment II, performance by 27 normal-hearing listeners on a novel speech comprehension test presented in noise was significantly associated with working memory capacity, but not with CSC. Moreover, this group showed no significant difference in CSC as the number of talker locations in the test increased. There was no consistent association between the CSC test and the RST. It is recommended that future studies investigate the psychometric properties of the CSC test, and examine its sensitivity to the complexity of the listening environment in participants with both normal and impaired hearing.

## Introduction

Participation in social activities has been found to be important for a person’s psychological and general well-being (Pinquart and Sörensen, 2000), and verbal communication is often the key to social interactions. Effective communication requires an interaction between implicit bottom–up and explicit top–down processes, and thus relies on both healthy auditory and cognitive systems (Wingfield et al., 2005; Pichora-Fuller and Singh, 2006; Schneider et al., 2010). Higher-level processing of speech, such as comprehension, inference making, gist formulation, and response preparation, involves in particular working memory processing (Daneman and Carpenter, 1980; Schneider et al., 2007; Wingfield and Tun, 2007). Working memory is defined as a limited capacity system with storage and processing capabilities that enables the individual to temporarily hold and manipulate information in active use as is necessary for comprehending speech (Baddeley, 1992; Just and Carpenter, 1992). In the widely accepted multi-component model of working memory, first introduced by Baddeley and Hitch (1974), the central executive is considered the control system for manipulation of input to either the phonological loop, visuospatial sketchpad, or episodic buffer (Repovš and Baddeley, 2006), and is considered the component that most influences working memory processing efficiency (McCabe et al., 2010). According to Miyake et al. (2000), the executive function is associated with three organizational processes; inhibition, shifting, and updating. When related to speech comprehension, these three processes refer to the ability to ignore irrelevant information, select the conversation to follow, and process the most recent sounds in order to compare items with stored knowledge to infer meaning, respectively.

Several speech perception models have been proposed to more specifically explain the mechanism of speech comprehension from sensory information, such as the cohort (Marslen-Wilson and Tyler, 1980; Marslen-Wilson, 1990), TRACE (McClelland and Elman, 1986; McClelland, 1991), and neighborhood activation (Luce and Pisoni, 1998) models. A more recent addition is the ease of language understanding (ELU) model (Rönnberg et al., 2008, 2013) that differs from the earlier models by its assumption that explicit working memory capacity is called for whenever there is a mismatch between the input signal and the phonological representations in long-term memory (Rönnberg et al., 2013). In brief, the ELU model stipulates the interaction between an implicit processing path and a slower explicit processing loop that run in parallel. While the multimodal input signal matches a sufficient number of phonological attributes in the mental lexicon, the lexical access proceeds rapidly and automatically along the implicit processing path with little engagement of the explicit processing loop. The explicit processing loop, which uses both phonological and semantic long-term memory information to attempt to understand the gist of the conversation, is, however, increasingly accessed when there is a mismatch between input signal and the phonological representations in long-term memory.

According to the ELU model, explicit working memory processing, including the executive processes, is increasingly relied on to infer meaning as the input signal becomes less clear and the listening situation more challenging. This notion is supported by several studies, which have shown that people with higher working memory capacity are less susceptible to distortion introduced by such factors as hearing impairment, increased complexity in the environment, or the introduction of unfamiliar signal processing in hearing devices; i.e., are better at understanding speech under such conditions (Lunner, 2003; Lyxell et al., 2003; Rudner et al., 2011a; Arehart et al., 2013; Meister et al., 2013). In these studies, a dual-task test, known as the RST (Daneman and Carpenter, 1980; Rönnberg et al., 1989), was used to measure the combined storage and processing capacity of working memory. The RST presents participants with a written set of unrelated and syntactically plausible sentences. After each sentence participants have to indicate if the sentence was sensible (e.g., the boy kicked the ball) or not (e.g., the train sang a song), and after a span of sentences they have to recall either the first or last word in the sentences (ignoring the article). Participants are presented with an increasingly longer span of sentences from 3 to 6. Performance on this paradigm has been found to be well-associated with speech comprehension (Daneman and Merikle, 1996; Akeroyd, 2008), and thus seems to be a solid predictor of inter-individual differences in speech processing abilities.

Recently, there has been an increased interest in the audiological community to prove that intervention with hearing devices, or specific device features, reduces cognitive resources allocated to listening; i.e., frees up resources for other cognitive processes such as higher-level speech processes (Sarampalis et al., 2009; Ng et al., 2013). This calls for an auditory test that taps into the cognitive functions engaged when communicating, such as working memory and the executive processes, and that is sensitive to different types of distortion and so can measure intra-individual differences in cognitive listening effort as the quality of the input changes. As one example of such a test, the concept of the RST was applied to the Revised Speech in Noise test to specifically investigate working memory capacity for listening to speech in noise (Pichora-Fuller et al., 1995). Using a mixture of high- and low-context sentences, participants were presented with a span of sentences and asked at the end of each sentence to indicate whether the final word was predictable from the sentence context or not, and at the end of the span to recall the final words. The authors found that age and increasing background noise disturbed the encoding of heard words into working memory, reducing the number of words that could be recalled.

New paradigms have also been introduced that aim to measure the CSC, defined as the residual capacity available for processing heard information after successful listening has taken place (Rudner et al., 2011b). An example is the CSCT, introduced by Mishra et al. (2013a), that taps into an individual’s working memory storage capacity, multimodal binding capacity (when visual cues are present), and executive skills after resources have been used for processing the heard stimuli. In this test participants are presented with lists of two-digit numbers, spoken randomly by a male or female talker, and are either asked to recall the highest (or lowest) numbers spoken by each talker, or to recall the odd (or even) numbers spoken by a particular talker. Thus the test measures the ability to update or inhibit information, respectively, and then recall the information, after resources have been spent on recognizing what has been said. The authors have argued that CSC as measured with the CSCT is different from general working memory capacity as measured with the RST. This is a reasonable assumption when considering the overall mental processes involved in the two tests. For example, the RST requires intake of written sentences, analysis of semantic content, formulation and delivery of a response, and storage and recall of words, whereas the CSCT requires attention to and processing of heard stimuli (potentially degraded by some form of distortion), a decision to be made about what to store, and storage, deletion, and recall of numbers. While there is some overlap in processes, there are also substantial differences, and therefore one would not expect a perfect correlation between performances on the two tests. Further, while reading the sentences in the RST for most people would be an implicit process, listening to the stimuli in the CSCT may require explicit processing as stipulated by the ELU model. That is, the CSC would be expected to be increasingly reduced under increasingly demanding listening conditions where explicit resources become involved in the processes of recognizing the input signal, leaving fewer resources for completing the remaining operations required by the CSCT. Therefore, it is likely that the residual capacity measured with CSCT under adverse test conditions is something less than the full working memory capacity measured with the RST. The authors of the CSCT have further suggested that during the updating or inhibition process of CSCT, if an executive resource that is required for performing these tasks has been depleted in the process of recognizing the numbers, the function of this particular resource may be at least partially compensated for by another cognitive resource that is separate from working memory. Consequently, a measure of working memory capacity may not adequately assess CSC. The CSCT has been evaluated with normal-hearing and hearing-impaired listeners under different conditions (Mishra et al., 2013a,b, 2014). Overall, the results, which are presented in more detail in the next section, suggested that the test has merit as a measure of cognitive listening effort. In addition, there was no overall association between CSCT and RST scores, suggesting that CSCT is not merely a measure of working memory capacity. In this paper we present an English version of the CSCT.

A hypothesis that a measure of CSC would better predict communicative performance than a measure of working memory capacity as captured with the RST (Mishra et al., 2013a) has not been investigated. Thus, we investigate in this paper if the CSCT or RST better predicts speech comprehension in noise. We recently developed and introduced a speech comprehension test that is designed to more closely resemble real world communication (Best et al., in review). This paradigm has been extended to include monologs and dialogs between 2 and 3 spatially separated talkers to study dynamic aspects of real communication. As the CSCT is designed to be administered under conditions similar to those in which speech performance is measured, it seems to provide an excellent tool for objectively investigating the cognitive effect of changing complexity of the listening conditions within individuals. We, therefore, further use the CSCT to investigate if dynamic changes in voice and location like those in our new speech test affect listening effort, as reflected in CSC.

In summary, this paper presents two experiments to address three aims. The aim of the first experiment is to present and evaluate an English version of the CSCT. The aims of the second experiment are to examine if CSC is a better predictor than working memory capacity of speech comprehension in noise, and to examine if increasing the number of talkers in the listening situation reduces CSC. In both experiments, listening conditions were simulated to represent, as best as possible, realistic listening environments. Treatment of test participants was approved by the Australian Hearing Ethics Committee and conformed in all respects to the Australian government’s National Statement on Ethical Conduct in Human Research.

## Experiment I

The aim of Experiment I was to evaluate an English version of the CSCT. The original Swedish test by Mishra et al. (2013a) was designed to measure both inhibition and updating. Different lists of thirteen two-digit numbers spoken randomly by a male and a female talker were made up for each task. For either task the listener was asked to remember at least two items. In the inhibition task, listeners were asked to remember the odd or even number spoken by one of the talkers, meaning they had to inhibit numbers spoken by the non-target talker. In the updating task, the task was to remember the highest or lowest number spoken by each talker, meaning that the listener had to update information stored in working memory when a new number met the criterion. Each list was designed to present three or four inhibition or updating events. A high memory load condition was created in which the listeners were further asked to remember the first number of the list, although this number was not taken into account in the final score.

In three studies, the Swedish version of the CSCT was evaluated by studying sensitivity to memory load (low vs. high), noise (quiet vs. stationary speech-weighted noise vs. modulated speech-like noise), and presentation modality (audio vs. audio-visual) in young normal-hearing and older hearing-impaired listeners (Mishra et al., 2013a,b, 2014). The older hearing-impaired listeners had stimuli amplified to compensate for their hearing loss, and for the noise conditions the SNR were individually selected to ∼90% recognition in the stationary noise. Overall, the studies showed that the older hearing-impaired listeners generally had reduced CSC relative to the younger normal-hearing listeners. For both populations, increasing the memory load and listening in stationary noise relative to quiet reduced CSC. Relative to quiet, the highly modulated speech-like noise reduced CSC in the older, but not in the younger cohort. The older hearing-impaired listeners also showed reduced CSC when listening in audio-only mode relative to audio-visual mode in noise and in quiet. Relative to the audio-visual mode, the younger normal-hearing listeners showed reduced CSC in audio-only mode when listening in noise, but increased CSC when listening in quiet. The authors argued that in all cases where CSC was relatively reduced, more pressures were put on the available cognitive resources needed for the act of listening, and that in the more demanding listening conditions visual cues counteracted for the disruptive effect of noise and/or poorer hearing (Mishra et al., 2013a,b, 2014).

In the studies conducted by Mishra et al. (2013a,b, 2014), task never interacted with any of the other factors, suggesting that the inhibition and updating measures were equally sensitive to different changes in the test condition. This is presumably because inhibition can be considered a part of the updating task, as items needed to be suppressed from working memory when a new item that fitted the criterion was stored. Consequently, to simplify the test design only the updating task was used in this study. The updating task was selected because the inhibition task in the Mishra studies generally produced higher scores than the updating task, with scores being close to ceiling for normal-hearing listeners. The decision to exclude the inhibition task meant that the need to switch between talker gender in the stimulus material was not strictly needed. There is a general belief that hearing-impaired people have more difficulty understanding female voices due to their more high-pitched characteristic (e.g., Helfer, 1995; Stelmachowicz et al., 2001), a factor that could have influenced the reduced CSC measured in the older hearing-impaired listeners by Mishra et al. (2014). To explore this further, we decided to present the updating task spoken by single talkers (one male or one female within each list), to test the effects of individual differences in talker characteristics (potentially including gender effects) on CSC. Removing the gender effect within lists meant that the listener did not have to attend to the talker gender during testing. On the other hand, the number of updating events in each list increased to four or five, with three lists introducing six updating events.

Like the Swedish version, the English version was further evaluated for sensitivity to population group (younger normal-hearing vs. older hearing-impaired listeners), noise (quiet vs. babble-noise vs. cafeteria noise), and presentation modality (audio only vs. audio-visual). While the Swedish test was evaluated under headphones with target and noise presented co-located, and in artificial noises, we chose to evaluate the CSCT under more natural listening conditions by presenting target and noise spatially separated in the free field, and using more realistic background noises. Introducing spatial separation in our presentation was expected to ease segregation (Helfer and Freyman, 2004; Arbogast et al., 2005), and hence the load on the executive function, for both normal-hearing and hearing-impaired listeners. However, this advantage was anticipated to be counteracted for during testing by choosing individual SNRs corresponding to the same speech recognition target used by Mishra et al. (2013b, 2014). Unlike the noises used by Mishra et al. (2013b, 2014) our babble- and cafeteria-noises were made up from intelligible discourses and conversations, respectively. As a result, our babble-noise was slightly more modulated than Mishra’s stationary noise, whereas our cafeteria-noise was slightly less modulated than Mishra’s speech-like noise. Finally, as in the Mishra studies, performance on the CSCT was related to measures of working memory capacity as measured with the RST and an independent test of updating. Overall, we expected to reproduce the findings by Mishra et al. (2014) with respect to the effect of population group, noise, and presentation modality, and we predicted that only the older hearing-impaired listeners would be affected by individual talker differences.

### Methodology

#### Participants

Participants included 11 females and 10 males recruited among colleagues and friends of the authors. Among the 21 participants, 12 could be considered younger normal-hearing listeners. Their average age was 31.6 years (ranging from 22 to 49 years), and their average bilateral 4FA HL, as measured across 0.5, 1, 2, and 4 kHz, was 0.4 dB HL (SE = 1.0 dB). The average age of the remaining nine participants was 72.3 years (ranging from 67 to 77 years), and they presented an average 4FA HL of 29.9 dB HL (SE = 3.0 dB). This group is referred to as older hearing-impaired listeners, although it should be noted that the hearing losses were generally very mild with the greatest 4FA HL being 46.3 dB HL. Participants were paid a small gratuity for their inconvenience.

#### The Stimuli

The stimulus material to measure CSC for updating was adapted from Mishra et al. (2013a). Audio-visual recordings of two-digit numbers were obtained using one male and one female native English speaker with Australian accents narrating the numbers 11–99 sequentially. Recordings were performed in an anechoic chamber, with the talkers wearing dark clothes and seated in front of a gray screen. Video recordings, showing head and shoulders of the talkers, were obtained using a Legria HFG10 Canon video-camera set at 1920 × 1080 resolution. Three high-powered lights were positioned to the sides and slightly in front of the talker, facing away from them and reflecting off large white surfaces, to smooth lighting of the face. Simultaneous audio recordings were obtained using a Sennheiser ME64 microphone, placed at close proximity to the mouth (about 35 cm), connected to a PC via a MobilePre USB M-Audio pre-amplifier. During recordings, the talkers were instructed to look straight ahead with a neutral expression, say the numbers without using inflection or diphthongs and close their lips between utterances. To ensure a steady pace, a soft beeping noise was used as a trigger every 4 s. Recording of the sequence of numbers was repeated twice for each talker.

The same set of 24 lists designed for the updating task was created for both the female and male talkers. To create the lists, the externally recorded audio was firstly synchronized to the video by aligning the externally acquired audio signal with the audio signal recorded with the video camera using a cross-correlation method in MATLAB. This technique can align two signals to an accuracy within 0.02 ms. Subsequently, the audio signal of each number was normalized in level to the same nominal value after removing gaps in the speech. A MATLAB program was then used to cut the long clips into short clips that were joined together according to the specified list sequences. For each number, the better of the two takes was used. The joined audio/video segments were cross-faded to ensure a smooth transition in both audio and video. In the final lists, the spoken numbers occurred roughly every 2.5 s. Finally, the audio was equalized per list to match the one-third octave levels of the ILTASS by Byrne et al. (1994).

**FIGURE 1:**
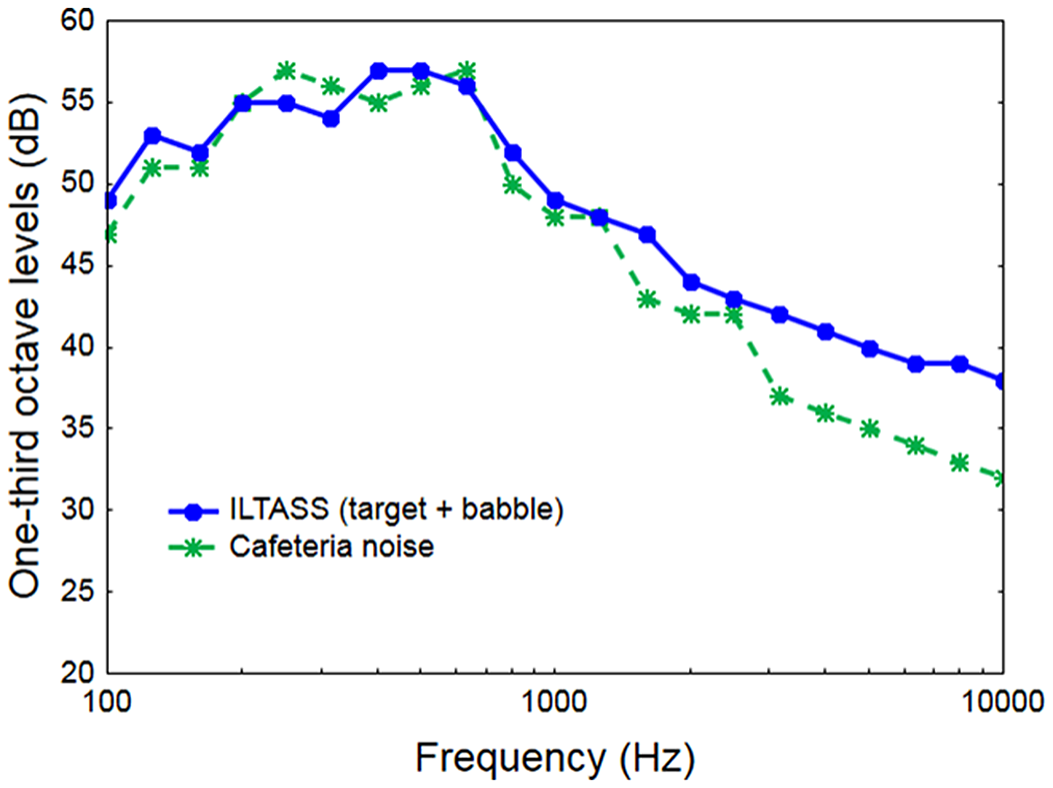
**The long-term spectra of the ILTASS (Byrne et al., 1994), that speech and babble-noise were filtered to match, and of the cafeteria-noise**.

Two kinds of background noise were used. One was an eight-talker babble noise from the National Acoustic Laboratories’ CDs of Speech and Noise for Hearing Aid Evaluation (Keidser et al., 2002). This noise had low amplitude modulation and was filtered to match the ILTASS. The other noise was a simulated reverberant cafeteria scene (for a detailed description of the scene, see Best et al., 2015). In brief, the noise was simulated such that the listener is positioned amongst the seating arrangements of a cafeteria with the target talker having a virtual position in the room in front of the listener. The background consists of seven conversations between pairs of talkers seated at the surrounding tables and facing each other, resulting in 14 masker talkers distributed around the listener at different horizontal directions, distances and facing angles. Room impulse responses generated in ODEON (Rindel, 2000) were converted to loudspeaker signals using a loudspeaker-based auralisation toolbox (Favrot and Buchholz, 2010). This noise was more amplitude modulated than the babble-noise, but not as modulated as single-talker speech. To maintain its natural acoustic characteristics, it was not filtered to match the target material. Consequently, when equalized to the same Leq, the cafeteria noise exposed the target at frequencies above 1.5 kHz, see **Figure 1**.

#### Setup

Speech and noise were presented spatially separated in the free field using a 16-loudspeaker array in the horizontal plane of the listener’s ears. The loudspeakers, Genelec 8020C active (self-amplified), were organized in a circle with a radius of 1.2 m and were driven by two ADI-8 DS digital-to-analog converters and an RME Fireface UFX interface, connected to a desktop PC. Using custom-made software, each loudspeaker was equalized (from 100 to 16000 Hz) and level-calibrated at the center of the array. The audio target was always presented from 0° azimuth at a level corresponding to 62 dB SPL at the position of the participant’s head. The video signal of the CSCT was shown on a 21.5 inch PC monitor mounted on an independent stand and appearing above the frontal loudspeaker. As the video was presented at a resolution of 1440 × 1080 to a monitor supporting a resolution of 1920 × 1080, a black bar occurred on either side of the video. Four uncorrelated samples of the babble-noise were presented from ±45° azimuth and ±135° azimuth, while the reverberant cafeteria-noise was played back from all 16 loudspeakers. Custom-made menu-driven software was used to mix and present target and noise at specified SNR values in a real-time fashion. While the long-term levels of both target and noise were controlled, the short-term SNRs were not to maintain a natural interaction between target and noise. That is, the audibility of individual numbers likely varied within and between participants. Across all presentations, the effect of this variation is presumed to be leveled out. For the hearing-impaired participants, amplification was applied to all stimuli following the NAL-RP prescription (Byrne et al., 1990), with gain tapered to 0 dB at frequencies above 6 kHz. The prescribed filters were applied in real-time to the combined target and noise stimuli.

#### Cognitive Tests

The English version of the RST was adapted from Hällgren et al. (2001) as an independent test of working memory capacity. Sentences were presented on a screen in three parts and in spans of 3–6 sentences. Within each span, the inter-sentence interval was 3000 ms. After the end of every sentence; i.e., every third screen, the participants were asked to say ‘yes’ or ‘no’ to indicate whether that sentence was sensible or not. At the end of each span the participants were asked to recall either the first or last word of the sentences in that span. After a practice trial, 12 spans of sentences were presented, increasing from three series of three sentences to three series of six sentences.

The Letter Memory test (Morris and Jones, 1990) was used as an independent test of updating. An electronic version of the test was developed that presents 320 point size consonants on a screen, one by one, for a duration of 1 s each. Participants were presented with sequences of 5, 7, 9, or 11 consonants, and asked at the end of each sequence to recall the last four consonants. After two practice trials, three trials of each sequence length were presented in randomized order.

#### Protocol

Each participant attended one appointment of about 2 h. First, the purpose of the study and the tasks were explained, and a consent form was signed. Otoscopy was performed, followed by threshold measurements. The participants then completed the RST and the Letter Memory test. Both tests were scored manually, with the final scores comprising the percentage of correctly recalled words and letters, respectively, irrespective of order. This part of the appointment took place in a regular sound-treated test booth.

The remaining part of the appointment took place in a variable acoustic room, adjusted to a reverberation time of T_60_ = 0.3 s. Participants were seated in the center of the loudspeaker array. First they completed an adaptive speech-in-noise test to determine the individual SNR for testing CSC in noise. Using the automated, adaptive procedure described in Keidser et al. (2013), sensible high context sentences (filtered to match the ILTASS) were presented in the eight-talker babble noise described above to obtain the SNR that resulted in 80% speech recognition. During the procedure the target speech was kept constant at 62 dB SPL while the level of noise was varied adaptively, starting at 0 dB SNR, based on the number of correctly recognized morphemes. Based on pilot data obtained on six normal-hearing listeners, the SNR was increased by 1 dB to reach the SNR that would result in ∼90% speech recognition when listening in babble-noise. This SNR was subsequently used in the CSCT with both the babble and cafeteria noises.

Finally, the CSCT was administered in a 2 (talker gender) × 3 (background noise, incl. quiet) × 2 (modality) design using two lists for each test condition. Test conditions were randomized in a balanced order across participants with lists further balanced across test conditions. After each list, participants had to recall either the two highest or the two lowest numbers in the list as instructed before each list. Because participants did not have to distinguish between talker gender while doing the updating task, a high memory load as introduced by Mishra et al. (2013a) was used; i.e., participants also had to remember the first number, as the task was otherwise considered too easy in the quiet condition for the younger normal-hearing listeners. The first number was not counted in the final score. During testing, participants verbalized their responses to the experimenter at the end of each list. Participants were instructed to look at the monitor during the audio-visual presentations, and this was reinforced by the experimenter who could observe the participants during testing. In the audio-only mode the video was switched off, meaning that the audio signal was the same in the two modalities.

### Results and Discussion

#### Reading Span and Updating Tests

**Table 1** lists the average performance data obtained by the two population groups on the reading span and updating tests. On both measures, the younger normal-hearing listeners outperformed the older hearing-impaired listeners. The differences in performance were significant according to a Mann–Whitney *U*-test (*p* = 0.0005 for the RST, and *p* = 0.03 for the updating test).

**Table 1:** Mean and SE values for RST and updating test for each population group.

	Young normal-hearing		Older hearing-impaired	
Parameter	Mean	SE	Mean	SE
RST (%)	49.4	3.02	32.0	2.22
Updating (%)	84.5	2.06	76.2	3.07

#### Test Signal-to-Noise Ratios

Individually selected SNRs were obtained for testing CSC in noise. On average, the older hearing-impaired listeners needed higher SNRs (-1.0 dB; SE = 0.6 dB) than the younger normal-hearing listeners (-4.5 dB; SE = 0.4 dB). The difference in mean was significant according to a Mann–Whitney *U*-test (*p* = 0.0001).

#### Cognitive Spare Capacity

**FIGURE 2:**
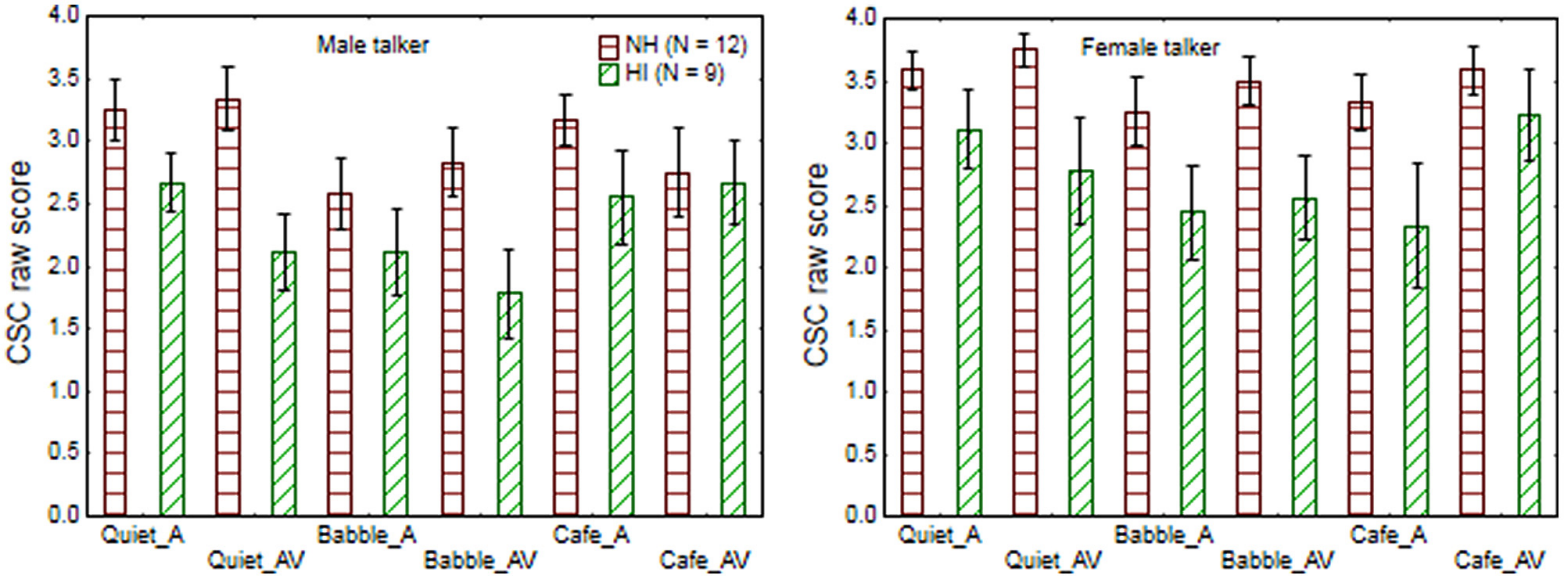
**The average CSC scores obtained by younger normal-hearing and older hearing-impaired participants when listening to a male talker (left graph) and female talker (right graph) in quiet, babble-noise (Babble), and in cafeteria-noise (Cafe) with audio-only (A) or audio-visual (AV) cues**.

**Figure 2** shows the average CSC score obtained by the younger and older listeners in each test condition. The arcsine transformed CSC scores were used as observations in a repeated measures ANOVA, using talker gender, noise, and modality as repeated measures and population group as grouping variable. This analysis revealed significant main effects of population group [*F*(1,19) = 11.5; *p* = 0.003], talker gender [*F*(1,19) = 11.6; *p* = 0.003], and noise [*F*(2,38) = 6.5; *p* = 0.004]. Specifically, the younger normal-hearing listeners showed more CSC than the older listeners across conditions, while CSC was reduced for the male talker (relative to the female talker) and by the presence of babble-noise (relative to quiet or cafeteria-noise). Modality did not show significance [*F*(1,19) = 0.6; *p* = 0.46], and none of the interactions were significant (*p*-levels varied from 0.08 for the three-way interaction of noise × modality × population group to 0.95 for the four-way interaction). Overall the English CSCT was sensitive to factors that could be expected to influence cognitive listening effort, although it differs from the Swedish CSCT by not showing sensitivity to presentation modality, and no significant interaction between noise, modality, and population group.

The English version of the CSCT differed from the Swedish version by having more updating events as a result of presenting all numbers by a single talker instead of switching between two talkers. Targets were further presented in the free field instead of under headphones. **Table 2** shows the differences in average scores obtained with the English and Swedish versions of CSCT for comparable test conditions. As there were no significant interactions with talker gender, the CSC scores obtained for the English test were averaged across talker gender, while the CSC scores obtained for the Swedish test were eyeballed off the graphs in Mishra et al. (2013b, 2014). Our results obtained in the audio-only mode compared well with the results on the Swedish version of the CSCT, suggesting that the modifications introduced to the actual test had negligible effects on CSC.

**Table 2:** The difference in CSC scores obtained for the English and Swedish samples (English – Swedish) on comparable test conditions with an updating task presented under high memory load.

	Audio-only mode	Audio-visual mode
**Normal-hearing**
quiet	-0.01	0.24
Noise with no or low modulation	0.06	0.13
Noise with high modulation	0.03	-0.21
**Hearing-impaired**
quiet	0.09	-0.87
Noise with no or low modulation	0.29	0.09
Noise with high modulation	-0.09	0.39

On the independent visual tests, the older hearing-impaired listeners showed significantly reduced updating skill and working memory capacity compared to the younger normal-hearing listeners. These findings are in agreement with MacPherson et al. (2002) who found that age has a negative association with performance on tests of executive function and working memory. The older hearing-impaired listeners also showed significantly reduced CSC compared to the younger normal-hearing listeners, which agrees with Mishra et al. (2014). The two groups differed in hearing loss as well as age. Hearing loss, even when aided, would impact on speech understanding because of distortions such as temporal processing (Fitzgibbons and Gordon-Salant, 1996; Gordon-Salant and Fitzgibbons, 2001). However, differences in the amount of speech understood (caused by differences in speech understanding abilities due to hearing loss as well as cognitive ability) were removed by using individually selected SNRs. Therefore, the finding suggests that aging effects observed in executive and working memory processing extend to CSC, or mental effort. This agrees with Gosselin and Gagné (2011) who found that older adults generally expended more listening effort than young adults when listening in noise under equated performance conditions.

Relative to the female talker, our participants, on average, showed reduced CSC when listening to the male talker. When comparing the two talker materials, the female talker was notably more articulate than the male talker. Thus the significant gender effect likely occurred because clear production of speech, rather than the female voice *per se*, freed up cognitive resources in the listeners. This is in agreement with observations of Payton et al. (1994) and Ferguson (2004, 2012) who found that both normal-hearing and hearing-impaired listeners performed better on nonsense sentences and vowel identification, respectively, when listening to a speaking style that was deliberately made clear relative to a conversational version. Further research with a range of male and female talkers is needed to fully explore the effect of talker gender on cognitive listening effort in older hearing-impaired listeners.

On average, our listeners showed a significant reduction in CSC when listening in the babble-noise relative to listening in quiet, which is in line with findings for a stationary noise by Mishra et al. (2013b, 2014). While the hearing-impaired listeners in Mishra et al. (2014) also showed a reduction in CSC relative to quiet when listening in a highly modulated speech-like background noise, the normal-hearing listeners did not (Mishra et al., 2013b). Mishra et al. (2013b) have suggested that the younger listeners could take advantage of a selective attention mechanism that comes into play when speech is presented against a speech-like noise (Zion Golumbic et al., 2013) to track the target speech dynamically in the brain. In the stationary noise, it was argued, the absence of modulations reduced the ability to track the speech. For the older listeners, their less efficient cognitive functions made it more difficult to separate the target speech from the non-target speech, whether the noise was modulated or not. An alternative way to view this is that speech understanding for the two groups was equated only in the unmodulated noise. As is well-known, hearing-impaired listeners are less able to take advantage of gaps in a masker (Festen and Plomp, 1990; Hygge et al., 1992; Peters et al., 1998), so in the modulated noise, the hearing-impaired listeners would have had to apply more cognitive resources than the normal-hearing listeners just to understand the speech. Consequently, the normal-hearing listeners were less likely to have had their cognitive capacity depleted by the modulated noise than was the case for the hearing-impaired listeners. Overall, findings on the two versions of CSCT suggest that both normal-hearing and hearing-impaired listeners expend executive resources on hearing out the target from a noise that has a similar spectrum and thus exerts a uniform masking effect across all speech components. In our study, neither population group showed significantly reduced CSC when listening in cafeteria-noise relative to quiet. The individually selected test SNRs were obtained in babble-noise, and it is possible that because the cafeteria-noise was more speech-like than the babble-noise, at the same SNR, spatial separation would in this case have an effect. This notion is supported by several studies that have demonstrated that when target and maskers are spatially separated, it is relatively easier to extract speech from the less than the more distinguishable masker (Noble and Perrett, 2002; Arbogast et al., 2005). In addition, it is possible that better SNRs at high frequencies available in our cafeteria-noise made speech easier to access (Moore et al., 2010). Combined, these two factors may have made it easier for both population groups to identify and track the target speech, and hence reduce the cognitive resources needed for understanding, especially as our hearing-impaired listeners had very mild hearing loss.

The main discrepancy between the Swedish and English version of the CSCT is that the Swedish version was sensitive to presentation mode while the English version was not. With the Swedish version, older adults generally showed more CSC in the audio-visual mode relative to the audio-only mode (Mishra et al., 2014), whereas younger adults showed this pattern in noise but the opposite pattern when listening in quiet (Mishra et al., 2013a,b). The authors argued that under more demanding listening situations, the addition of visual cues counteracted the disruptive effect of noise and/or poorer hearing. This argument is supported by Frtusova et al. (2013) who found that visual cues facilitate working memory in more demanding situations for both younger and older adults, and Fraser et al. (2010) who saw a reduction in listening effort when introducing visual cues in a dual-task paradigm involving listening to speech in noise. For the younger cohort, the authors speculated that while listening in quiet, the auditory processing task was implicit, meaning that the visual input became a low priority stimulus and hence a distractor (Lavie, 2005), such that audio-visual integration required in the audio-visual mode added demand to the executive processing capacity. No effect of modality was observed in this study, which could suggest that our test conditions were not as cognitively demanding as those used by Mishra and colleagues although the data obtained in the audio-only mode in **Table 2** seem to refute this theory. Another possible reason for the lack of a visual effect in our study is poor attention to the video signal (Tiippana et al., 2004). Although the participants were all looking directly at the screen during testing, the room in which testing was conducted presented a lot of distracting visual information, including colorful wall panels, and the array of loudspeakers and other test equipment. Lavie (2005) has demonstrated that even when people have been specifically instructed to focus attention on a visual task, they are easily distracted while the perceptual load in the visual modality is low. Other data on the association between audio-visual integration and executive function are divided (Prabhakaran et al., 2000; Allen et al., 2006), hence, the visual effect on CSC needs a more systematic investigation.

#### The Association between CSC and Other Cognitive Measures

Regression analyses were performed to investigate the association between the factor-wise CSC scores (i.e., scores averaged across various experimental conditions) obtained on all participants and the other two cognitive measures, when either controlling for 4FA HL or age. Separate regression analyses were performed using each of the reading span and updating measures as independent variable. The results are summarized in **Table 3**. In all cases, the regression coefficient was positive, sometimes significantly so; suggesting that more CSC was associated with better cognitive function. The results were little affected whether age or hearing loss was used as the co-variate. In agreement with Mishra et al. (2013a,b, 2014), the CSCT was more strongly related to the updating test than to the RST. Overall, the more consistent association with the independent updating test and inconsistent association with the RST suggest that the CSCT measures something more similar to the combination of attributes used in the updating task than those used in the RST. However, for none of the individual CSC scores is the association between CSC and updating skill significantly greater than the association between CSC and reading span measures. We further note that moderate, but significant, correlations have been found between measures of memory updating and complex working memory spans (e.g., Lehto, 1996).

**Table 3:** The standardized regression coefficients (ß) and their SE values related to the extent to which CSC scores are predicted by performance on the RST or updating test when controlling for degree of hearing loss (4FA HL) or age.

	RST (%)				Updating test (%)			
	4FA HL (dB HL)		Age (year)		4FA HL (dB HL)		Age (year)	
Parameter	ß	SE of ß	ß	SE of ß	ß	SE of ß	ß	SE of ß
CSCT overall	0.50	0.23	0.38	0.23	0.59^∗∗^	0.18	0.54^∗∗^	0.18
Male	0.59^∗^	0.25	0.54^∗^	0.23	0.60^∗∗^	0.18	0.60^∗∗^	0.19
Female	0.30	0.29	0.15	0.24	0.45^∗^	0.21	0.38	0.20
Quiet	0.17	0.27	0.13	0.23	0.57^∗∗^	0.17	0.51^∗∗^	0.17
Cafeteria	0.70^∗^	0.26	0.52	0.25	0.56^∗^	0.20	0.55^∗^	0.21
Babble	0.43	0.27	0.34	0.23	0.41^∗^	0.20	0.36	0.20
A-only	0.26	0.30	0.11	0.25	0.54^∗^	0.20	0.48^∗^	0.20
AV	0.65^∗^	0.23	0.58^∗^	0.20	0.55^∗∗^	0.17	0.53^∗^	0.18

One asterisk indicates a significance level <0.05, and two asterisks a significance level <0.01.

## Experiment II

The aims of Experiment II were to examine, in normal-hearing listeners, if CSCT or RST measures would better predict comprehension of dynamic conversations, and if CSC is reduced when increasing the dynamics of the listening situation. Speech performance was measured using a new speech comprehension test that delivers monologs and conversations between 2 and 3 spatially separated talkers. Participants listened to the speech and answered questions about the information while continuing to listen. To parallel the dynamic speech comprehension test, the CSCT stimuli were presented either all from a single loudspeaker position, or randomly from two or three loudspeaker positions. Both the CSCT and the dynamic speech comprehension test were implemented under realistic acoustic conditions in a cafeteria background.

Considering the mental processes involved in performing the RST (reading words, deriving meaning from the words, forming and delivering a response, storing items, and recalling items), the CSCT (segregating target speech from noise, recognizing the words, making decision about what to store, storing items, deleting items, and recalling items), and the speech comprehension test (segregating target speech from noise, recognizing, the words, deriving meaning from the words, storing items, recalling items and forming and delivering a response,), it would seem that the speech comprehension test shares processes with both the RST and the CSCT, and that only a couple of operations are common to all three tests. Based on a comparison of the mental processes the pairs of tests have in common, it could be expected that speech comprehension performance would be more correlated with performance on the RST if individual differences in the ability to process words to derive meaning and form a response are more important in causing individual differences in speech comprehension than individual differences in identifying which speech stream is the target, segregating it, and recognizing the words. With our group of normal-hearing listeners we expected the former to be the case and hence we predicted performances on our comprehension test to be associated more strongly with RST than with CSCT measures. We further expected that increasing the dynamic aspects of speech by changing voice and location of talkers more frequently would add processing demands in working memory, and in the executive function specifically, so that the listeners would require better SNRs to perform as well in the conversations as in the monologs (Kirk et al., 1997; Best et al., 2008), and that between listening conditions, variations in the CSC would be correlated with variations in speech comprehension.

### Methodology

#### Participants

The participants were primarily university students and included 16 females and 11 males. All had normal hearing, showing an average 4FA HL of 2.9 dB HL (SE = 0.6 dB). The age of the participants ranged from 18 to 40 years, with an average of 26.2 years. Participants were paid a small gratuity for their inconvenience.

#### Dynamic Speech Comprehension Test

The dynamic speech comprehension test consists of 2–4 min informative passages on everyday topics that are delivered as monologs or conversations between two or three talkers. The passages are taken from the listening comprehension component of the International English Language Testing System, for which transcripts and associated comprehension questions are publicly available in books of past examination papers (Jakeman and McDowell, 1995). The recorded presentations are spoken by voice-actors who were instructed to read the monologs and play out the conversations in a natural way, including variations in speed, pauses, disfluencies, interjections etc. Each passage is associated with 10 questions that are answered “on the go” (brief written responses) while listening.

#### Setup

Testing took place in an anechoic chamber fitted with 41 equalized Tannoy V8 loudspeakers distributed in a three-dimensional array of radius 1.8 m. In the array, 16 loudspeakers were equally spaced at 0° elevation, eight at ±30° elevation, four at ±60° elevation, and one loudspeaker was positioned directly above the center of the array. Stimuli were played back via a PC equipped with an RME MADI soundcard connected to two RME M-32 D/A converters and 11 Yamaha XM4180 four-channel amplifiers.

Testing was done in a simulated cafeteria scene similar to that used in Experiment I. The background noise was simulated using ODEON software (Rindel, 2000) in the same way as described for the cafeteria noise in Experiment I, but using different room characteristics, and the entire 41 loudspeaker array. As previously, the background of the cafeteria noise consisted of seven conversations between pairs of talkers seated at tables and facing each other, resulting in 14 masker talkers distributed around the listener at different horizontal directions, distances and facing angles. The listener was situated by a table slightly off center in the room, facing three talkers positioned 1 m away at -67.5, 0, and +67.5° azimuth. During testing, monologs were presented from either of these three loudspeaker locations. For the two-talker condition, conversations took place between talkers situated at -67.5 and 0°, at 0 and +67.5°, or at -67.5 and +67.5° azimuths. The three-talker conversations all involved the talkers at each of the three loudspeaker locations. While speech was presented from each of these loudspeakers, an LED light placed on top of the loudspeaker was illuminated to give the listener a simple visual cue to indicate which source was active, as would be indicated by facial animation and body language in a real conversation.

#### Protocol

Each participant attended three appointments of about 2 h. During the first appointment, the purpose of the study and the tasks were explained, and a consent form was signed. Otoscopy was performed, followed by threshold and reading span measurements. The implementation of the RST was the same as used in Experiment I. The dynamic speech comprehension test was completed over the three appointments, and the CSCT was administered at either the second or third appointment.

For the dynamic speech comprehension test, the target speech was fixed at 65 dB SPL and all participants were tested in each talker condition at three SNRs (-6, -8, and -10 dB), using five passages (i.e., 50 scoring units) for each SNR. The participant was seated in the anechoic chamber such that the head was in the center of the loudspeaker array, facing the frontal loudspeaker. Note that participants were allowed to move their head during testing to face the active source. Responses were provided in written form using paper and pencil and scored manually post-testing. The different passages were balanced across test conditions, and talker conditions and SNRs were presented in a randomized order across participants. The source position of the talkers also varied randomly across and within passages.

The CSCT was presented in a similar fashion to the dynamic speech test at a -6 dB SNR. Three lists were administered for each talker condition and the combined score obtained. To parallel the one-talker condition, one list was presented from each of the three talker locations (-67.5, 0, and +67.5° azimuths). To parallel the two-talker condition, numbers were for one list randomly presented from -67.5° and 0° azimuths, for another list randomly presented from 0 and +67.5° azimuths, and for the final list randomly presented from -67.5 and +67.5° azimuths. To parallel the three-talker condition, numbers for each of the three lists were randomly presented from the three loudspeaker locations. To reduce the chance of reaching ceiling effects, a high memory load was implemented by asking the participants to also recall the first number in each list, although the number was not counted in the final score. Before CSC testing, one list was presented in -6 dB SNR, with numbers coming randomly from two loudspeaker locations, and participants were asked to repeat back the numbers heard. One missed number was allowed; otherwise the SNR was increased to ensure that the participants were able to hear the numbers in the noise. No participants needed the SNR changed. Nine lists from a pool of 12 were randomly selected for each participant and randomly presented across talker condition and locations.

### Results and Discussion

#### Speech Comprehension

**FIGURE 3:**
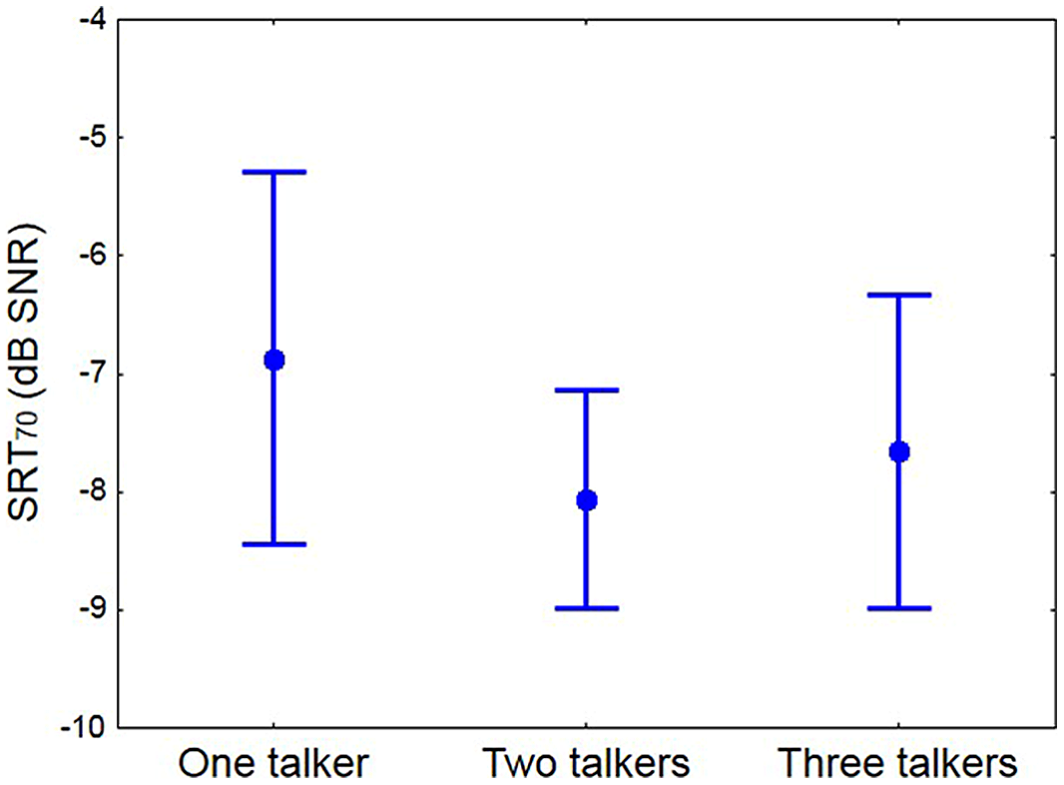
**The mean SRT_70_ for each talker condition. Whiskers show 95% confidence interval**.

For each participant a logistic function was fitted to the three data points measured with the comprehension test for each talker condition, and the SNR for 70% correct answers was extracted (SRT_70_). For three participants, the data obtained for one talker condition (single-talker or three-talker) were not well-behaved as a function of SNR, and thus sensible logistic functions could not be fit. From the remaining 24 participants, the average differences in SRT_70_ between the 1- and 2-talker, and between the 2- and 3-talker conditions, were obtained. These differences were applied as appropriate to the two-talker SRT_70_ values measured for the three participants with missing data points to obtain extrapolated replacement values. According to a repeated measures ANOVA the difference in SRT_70_ between talker conditions was significant [*F*(2,52) = 3.92; *p* = 0.03], **Figure 3**. A Tukey HSD *post hoc* analysis revealed that the listeners required significantly higher SNRs to reach 70% correct scores on the monologs than on the dialogs. We note that the ranking of conditions in terms of SRTs corresponds to the complexity of the language of the passages, as measured with the Flesch–Kincaid Grade level (Kincaid et al., 1975; 9.7, 3.5, and 6.1 for the one, two, and three-talker passages, respectively). This suggests that speech comprehension may be more affected by complexity of the spoken language, in terms of length and number of words used, than by the dynamic variation in talker location.

#### The Sensitivity of CSC to Increased Dynamic Variation

**FIGURE 4:**
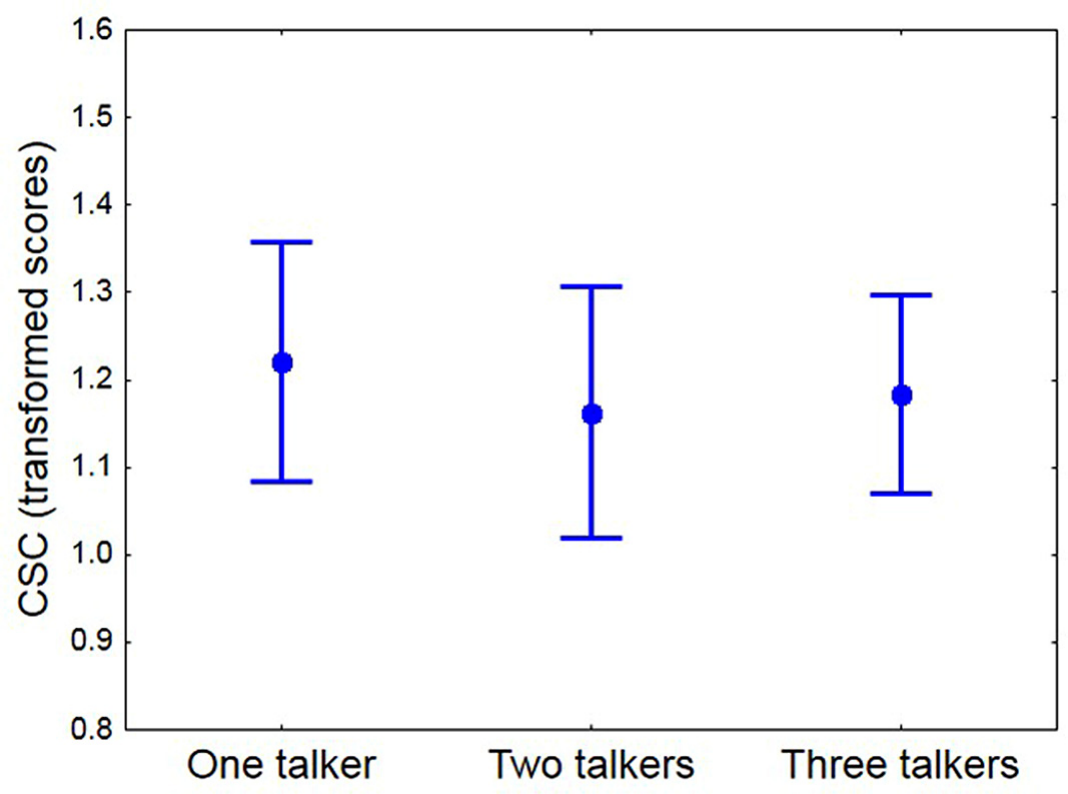
**The mean transformed CSC score for each simulated talker condition (maximum = 1.57). Whiskers show 95% confidence interval**.

To investigate if CSC was affected by increasing the number of talkers in the listening situation, the combined scores across three CSC lists were obtained for each participant and simulated talker condition. Based on arcsine transformed scores, participants, on average, showed slightly reduced CSC for the simulated two-talker condition relative to the simulated one- and three-talker conditions, **Figure 4**. According to a repeated measures ANOVA this pattern was not significant [*F*(2,52) = 0.27; *p* = 0.76], suggesting that, at least for younger normal-hearing listeners, increasing the complexity of the listening condition, by increasing the number of target locations, did not reduce CSC. It is worth noting, that the lowest average CSC of 1.1 transformed scores was obtained for the two-talker condition in which the target locations were most separated (by 67.5°).

#### Predicting Inter-Participant Variation in Speech Comprehension

Across participants, reading span scores varied from 28 to 70% with a mean of 45.5%. This result is not unlike findings by Zekveld et al. (2011), who reported a mean reading span score of 48.3%, ranging from 30 to 74%, on a slightly younger normal-hearing sample. **Table 4** lists the correlation coefficients for the associations between reading span scores and transformed CSC scores obtained for each talker condition (first column). Reading span scores were positively and significantly associated with the transformed CSC scores obtained for the simulated two-talker condition (*p* = 0.03), but not for the simulated one- and three-talker conditions (*p* = 0.83 and *p* = 0.69, respectively). The fact that CSC scores are not consistently correlated with reading span measures across all three conditions may suggest again that the two tests do not generally capture the same cognitive constructs, although none of the correlation coefficients were significantly different from each other.

**Table 4:** The correlation coefficients and their 95% confidence intervals (shown in brackets) for associations of interest between RST, CSCT, and SRT_70_ measures.

Parameter	RST	SRT_70_ (1-talker)	SRT_70_ (2-talker)	SRT_70_ (3-talker)	SRT_70_ (collapsed)
RST		-0.51** [0.15,-0.75]	-0.52** [-0.17,-0.76]	-0.57** [-0.24,0.78]	-0.57** [-0.24,-0.78
CSCT (1-talker)	0.04 [0.42,-0.35]	-0.05 [0.34,-0.43]			
CSCT (2-talker)	0.4* [0.69,0.04]		-0.29 [0.11,-0.61]
CSCT (3-talker)	-0.08 [0.32,-0.45]			0.09 [0.46,-0.31]
CSCT (collapsed)	0.21 [0.55,-0.19]				-0.21 [0.19,-0.55]

One asterisk indicates a significance level < 0.05, and two asterisks a significance level < 0.01.

To determine whether CSCT or RST best predicted inter-participant variation in speech comprehension, correlation coefficients for the association between reading span scores and performance on the speech comprehension test in each talker condition (first row), and for each talker condition the association between transformed CSC scores and performance on the speech comprehension test were obtained, see **Table 4**. For all three talker conditions, data suggest that good performance on the dynamic speech comprehension test requires good working memory capacity (*p* < 0.01 for all three talker conditions), but is not significantly associated with cognitive listening effort as measured with the CSCT (*p* = 0.82, *p* = 0.15, and *p* = 0.67 for the one-, two-, and three-talker condition, respectively). As associations between measures were consistent across talker conditions, data for the CSCT and speech comprehension measures were further collapsed across talker conditions to do an overall three-way correlation analysis. As can be seen in **Table 4**, the association between RST and the collapsed SRT_70_ is highly significant (*p* = 0.002), while the association between the collapsed CSC and SRT_70_ is not (*p* = 0.30). The difference between the correlation coefficients obtained for the two associations is, however, not significant (*p* = 0.13), meaning that no strong conclusion can be made about the relative strengths of the associations. Looking at the three-way correlation matrix, where the association between the collapsed CSC scores and RST is also non-significant (*p* = 0.31), it is evident, however, that the strongest similarity is found between the SRT_70_ and RST measures.

## Overall Discussion

Two experiments were presented in this paper. In the first experiment we evaluated an English version of the CSCT introduced by Mishra et al. (2013a) that focuses on measuring an individual’s CSC for updating processing after processing of auditory stimuli has taken place. In the second experiment we investigated if this measure of CSC or a measure of working memory capacity, using the RST, better predicted variation in speech comprehension, and if CSC was reduced when increasing the number of talkers in the listening situation.

In agreement with Mishra et al. (2013a,b, 2014) we found in both experiments indications that the CSCT measures a construct different from the RST. This was expected as the two test paradigms do differ in some of the mental processes that are required to perform the specific tasks of the tests. The evidence was, however, not strong. Specifically, we note that with an administration of two lists per test condition, 74% of variance in CSC scores obtained in Experiment I was due to intra-participant measurement error variance, which would have reduced the reported regression coefficients. Further, there is some concern to what extent participants actively engage in updating when the task is to recall the last items in a list of an unknown number of items, as is the case in the independent updating task employed in Experiment I, or whether they simply wait until the end of the list before attempting to recall the most recent items (Palladino and Jarrold, 2008). Consequently, the correlation analyses presented in this study and in Mishra et al. (2013b, 2014) on the associations between the RST and the CSCT scores and between the independent updating task and the CSCT scores should be interpreted with caution. Overall, it would be desirable in the future to establish the psychometric properties of the CSCT, including determining the ideal number of lists for reliable measures of CSC, and to more systematically explore the relationship between CSCT, RST, and other tests of executive processing and working memory capacity.

Evaluated in a more natural listening environment than that used by Mishra et al. (2013a,b, 2014), we confirmed in Experiment I that the CSCT has merit as a concept for measuring the cognitive effort associated with listening to speech that has been degraded by some form of distortion. Specifically, we found that the CSCT was sensitive to population group and a masker with low modulation (relative to listening in quiet), and further to clarity of speech. On the other hand, we could not confirm in Experiment I that CSC is affected by a masker with high modulation in hearing-impaired listeners or by presentation modality in either population group. Methodological variations are suggested to account for the differences observed between the English and Swedish version of the CSCT. Specifically, spatial separation of target and masker, and exposure to high-frequency speech energy when listening in the highly modulated cafeteria-noise likely made it easier for both population groups to access and track target speech (Arbogast et al., 2005; Moore et al., 2010), and hence in line with the ELU model made this test condition less taxing on cognitive effort. A low perceptual load in the visual modality and distracting visual information in the test environment were suggested to combine to have made participants prone to relax their attention to the video signal (Tiippana et al., 2004; Lavie, 2005), to reduce its potential effect on cognitive listening effort. It would be of interest to study these factors more closely in the future. It should also be noted that if our implementations indeed were closer to real-life listening, this study would suggest that cognitive listening effort may not be as easily modulated by the listening condition in real life as demonstrated in some laboratory tests.

As predicted on the basis of the mental processes involved in our speech comprehension test, and our participant sample having normal-hearing, we found in Experiment II that those with poorer working memory capacity required better SNRs to perform at a similar level on the comprehension test than those with greater capacity. The association between speech comprehension and working memory capacity was significant, while the association between speech comprehension and CSC was not, suggesting that individual differences in speech comprehension may be more related to individual abilities to process words to derive meaning and form a response than to the individual abilities to overcome the perceptual demand of the task. This finding ties in well with the established association between span tests, such as the RST that tap into the combined processing and storage capacity of working memory, and speech comprehension (Daneman and Merikle, 1996; Waters and Caplan, 2005), and further lends support to the ELU model. We speculate, however, that we may see an opposite trend in a hearing-impaired population; i.e., find a significant association between speech comprehension and CSC instead. This is because the individual abilities in this population to meet the perceptual demands of the CSCT may outweigh the variation in individual abilities to process written words to derive meaning and form a response.

The finding in Experiment II that increasing the dynamic variation in voice and location from 1 to 2 and three talkers did not systematically affect speech comprehension performance in young normal-hearing participants, when they listened in a reverberant cafeteria-like background, was somewhat surprising. We had expected that the participants would have required slightly better SNRs for comprehending speech when listening to more than one talker (Kirk et al., 1997; Best et al., 2008) as turn-taking becomes less predictable, increasing the challenge of identifying the current talker and monitoring and integrating what each talker said. That is, they needed to expend more cognitive resources when listening to the conversations. However, it is possible that the increased cognitive demand arising from applying attention to location was counteracted by advantages from having a greater number of discourse markers and more informative perspectives from multiple talkers in the multi-talker conversations (Fox Tree, 1999). A significantly higher SRT_70_ measured for monologs than for dialogs may be explained by more and longer words being presented in the monologs than in the two-person conversations. This finding is in line with other studies that have seen sentence complexity impacting on speech comprehension performances (Tun et al., 2010; Uslar et al., 2013). The theory is also supported by findings that longer words reduce memory spans of sequences of words (Mueller et al., 2003); i.e., demand more working memory processing. However, we saw no difference in the strengths of the associations between RST scores and speech comprehension across talker conditions (cf. **Table 4**).

Previous studies have shown that measures of cognitive effort can be more sensitive to subtle changes in the listening situation than measures of speech understanding (e.g., Sarampalis et al., 2009; Ng et al., 2013). Thus, we expected that the CSCT might be sensitive to dynamic variations in target location even where our comprehension task was not. However, we found in Experiment II that applying random dynamic variations to the speech targets of the CSCT did not generally lead to reduced CSC in our normal-hearing participants, although it is of interest that the average lowest CSC was observed for the condition when numbers were presented randomly from the two most distant locations. Despite using transformed CSC scores in our analysis, our result may be partly influenced by many listeners reaching ceiling on the CSCT across test conditions (35% of total scores). It is also possible that allowing listeners to naturally move their head to listen to the spatially separated targets reduced differences in CSC, especially when distances between target locations were less extreme. On the other hand, it appeared from spontaneous comments that at least for some participants the shifting location of the target did not interfere with the task of updating the heard input, and thus it is possible that dynamic changes in target location did not actually represent a change in difficulty. It is worth noting that in the CSCT the actual voice did not change with location as it did in the dynamic speech comprehension test.

Future studies in our laboratory will further investigate to what extent CSC is sensitive to increasing complexity in the environment, and will also examine the effect of age and hearing loss on associations between CSC and the listening environment.

## Conflict of Interest Statement

The authors declare that the research was conducted in the absence of any commercial or financial relationships that could be construed as a potential conflict of interest.

## Abbreviations

4FA HL: four-frequency average hearing loss
ANOVA: analysis of variance
CSC: cognitive spare capacity
CSCT: cognitive spare capacity test
ILTASS: international long-term average speech spectrum
RST: reading span test
SE: standard error
SNR: signal-to-noise ratio
SRT: speech reception threshold.
